# Influence of Fermentation of Pasteurised Papaya Puree with Different Lactic Acid Bacterial Strains on Quality and Bioaccessibility of Phenolic Compounds during *In Vitro* Digestion

**DOI:** 10.3390/foods10050962

**Published:** 2021-04-28

**Authors:** Florence M. Mashitoa, Stephen A. Akinola, Vimbainashe E. Manhevi, Cyrielle Garcia, Fabienne Remize, Retha. M. Slabbert, Dharini Sivakumar

**Affiliations:** 1 Department of Horticulture, Tshwane University of Technology, Pretoria West 0001, South Africa; mashitoakganase@gmail.com (F.M.M.); slabbertmm@tut.ac.za (R.M.S.); 2 Phytochemical Food Network Group, Department of Crop Sciences, Pretoria West 0001, South Africa; akinolastephen3@gmail.com (S.A.A.); manhivive@tut.ac.za (V.E.M.); 3 Qualisud, Univ Montpellier, Univ de La Réunion, CIRAD, Institut Agro, Avignon Université, F-34398 Montpellier, France; cyrielle.garcia@univ-reunion.fr (C.G.); fabienne.remize@univ-reunion.fr (F.R.)

**Keywords:** postharvest preservation, *Lactobacillus*, antioxidant activity, polyphenols, *in vitro* digestion

## Abstract

This study describes the impact of utilising different strains of lactic acid bacteria (LAB) for the fermentation of papaya puree and their effect on the quality parameters and bioaccessibility of phenolic compounds during simulated *in vitro* gastrointestinal digestion. Papaya was processed into puree; pasteurised and fermented at 37 °C for 2 days; and stored for 7 days at 4 °C using LAB strains *Lactiplantibacillus plantarum 75* (L75*D2; L75*D7), *Weissella cibaria* *64* (W64*D2; W64*D7) and *Leuconostoc pseudomesenteroides 56* (L56*D2; L56*D7), respectively. Non-fermented samples at 0 (*PPD0*), 2 (*PPD2*) and 7 days (*PPD7*) served as controls. pH was reduced with fermentation and was lowest in L56*D2 (3.03) and L75*D2 (3.16) after storage. The colour change (*ΔE*) increased with the fermentation and storage of purees; L75*D7 showed the highest *ΔE* (13.8), and its sourness reduced with storage. The fermentation by W64*D7 and L75*D7 increased the % recovery of chlorogenic, vanillic, syringic, ellagic, ferulic acids, catechin, epicatechin and quercetin in the intestinal fraction compared to the L56*D7 and *PPD7*. Fermentation by W64*D7 and L75*D7 significantly improved the antioxidant capacity of the dialysed fraction compared to the L56*D7 or *PPD7*. L56*D7-fermented papaya puree showed the highest inhibitory effect of α-glucosidase activity followed by L75*D7. L75*D7 had a significantly higher survival rate. LAB fermentation affected the bioacessibilities of phenolics and was strain dependent. This study recommends the use of *Lpb. plantarum 75* for fermenting papaya puree.

## 1. Introduction

Regular consumption of fruits and vegetables is important for a healthy lifestyle, and for the reduction in risk factors for non-communicable diseases [1]. Reports from the World Health Organization (WHO) and Food and Agriculture Organization (FAO) [2] of the United Nations in 2004 recommended an intake of 400 g of fruit and vegetable per day. However, the highly perishable nature of fruits and vegetables and lack of cold chain facilities, coupled with the energy cost requirements, limit their availability and shelf life. Therefore, fermentation technology guarantees the availability and safety of fruits and vegetables to the consumers during the off season [3]. Several researchers have reported an improvement in the antioxidants of fruits and vegetables during lactic acid fermentation [4,5].

*Carica papaya* Linn., belonging to the family *Caricaceae*, known as pawpaw or papaya, is popularly produced and consumed in South America, Asia and Africa [6]. Papaya is a rich source of carotenes, vitamin C, flavonoids, antioxidants, folate, potassium, magnesium and fibre [6]. LAB fermentation rapidly reduces the pH, thus increasing acidity, which prevents the spoilage of fermented products [7]. Furthermore, fermentation enhances the antioxidant properties through the biotransformation of phenolic compounds by the metabolising LAB strains, resulting in the release of bioactive compounds [8]. However, the potential functionality of a compound depends on the amount that is available after gastrointestinal digestion compared to the original amount before digestion. The pH changes that occur during gastrointestinal digestion phases produce phenolic derivatives that are high in molecular weight, have low solubility and are unavailable for absorption, mainly due to oxidation or polymerisation reactions [9]. Shahid and Peng [10] stated that the interaction with protein, lipid, fibre and hydrolytic enzymes affects the bioaccessibility of phenolic compounds in the intestinal tract. *In vitro* digestion models are widely used by researchers to mimic digestion due to it cost effectiveness [9] and non-ethical clearance requirement. Pavan et al. [11] showed changes in total phenols and antioxidant activity during gastrointestinal digestion before and after the digestion of tropical fruits, such as araticum, papaya and jackfruit, and digestion decreased the levels of total phenols and antioxidant activity in papaya extracts. However, their finding did not show the influence of gastrointestinal digestion on predominant different phenolic components present in papaya juice. Therefore, we hypothesise that the fermentation with LAB increases the total phenol content, different phenolic metabolites and antioxidant capacity in fermented papaya puree after digestion at the dialysis phase, which is available for intestinal absorption. 

The total phenolic content in papaya is reportedly 54 mg GAE/100 g fresh weight (FW) [12]; however, the concentrations differ in various cultivars. Gayosso-García Sancho et al. [13] reported the ferulic acid content as 277.49–186.63 mg/100 g dry weight (DW), p-coumaric acid (229.59–135.64 mg/100 g DW) and caffeic acid (175.51–112.89 mg/100 g DW) contents in Maradol papaya from Mexico. Phenolic compounds inhibit α-glucosidase and reduce glucose uptake in the small intestine, through the inhibition of disaccharide digestion [14]. A commercial fermented papaya preparation sold in Japan and the Philippines reportedly showed a significant decrease in plasma glucose levels in type 2 diabetic patients [15]. Therefore, this study was aimed to investigate the effect of LAB fermentation on the quality parameters, changes in major phenolic compounds, α-glucosidase activity and to evaluate the influence of *in vitro* gastrointestinal digestion on phenolic components and antioxidant capacity of the pasteurised and fermented papaya puree. 

## 2. Materials and Methods

### 2.1. Chemicals

Culture media were purchased from Biokar Diagnostics (Solabia group, Pantin, France) and Conda Laboratories (Madrid, Spain). Reagents were obtained from Sigma-Aldrich (Saint-Quentin Fallavier, France) and VWR chemicals (Fontenay-sous-Bois, France). Type VI-B porcine pancreatic α-amylase, type I α-glucosidase from baker’s yeast, starch, p-nitrophenyl-β-glucopyranoside (pNPG) and voglibase and other chemicals came from Sigma-Aldrich (Saint-Quentin Fallavier, France).

### 2.2. Preparation of Fruit Purees 

The purchase of fruits was from the local growers in Réunion Island. Purees were prepared by peeling, cutting and blending the fruit pieces. The bottled fruit purees were then pasteurised in an agitating water bath at 80 °C for 15 min and cooled to room temperature (28 °C) for 2 h prior to fermentation. 

### 2.3. Reactivation of LAB Cultures and Fermentation of Fruit Purees

Lactic acid bacteria used in the study were previously isolated from tomatoes (*Lycopersicon esculantum*), papaya (*Carica papaya*) and sliced cabbage (*Brassica oleacera var. capitata*) and genotyped and have been reported as safe [3]. The LAB strains *Leuconostoc pseudomesenteroides 56, Weissella cibaria 64* and *Lactiplantibacillus plantarum 75* were reactivated at 30 °C for 48 h in de Mann Rogosa Sharpe (MRS) broth (Biokar Diagnostics, Pantin, France). 

An aliquot of 100 μL of each culture was transferred into new 9 mL MRS broth and then incubated for 48 h at 30 °C. Repeating the reactivation step twice was to achieve an active growing condition of the cultures, after which the culture cells were produced anaerobically in MRS broth incubated at 30 °C for 24 h. The LAB cell pellet, obtained after centrifugation at 8000× *g* for 5 min, was washed twice with sterile distilled water. The resulting LAB cells, re-suspended in 20 mL of sterile water, were used as stock of concentrated LAB cultures. The concentrations of the LAB cultures were determined using a spectrophotometric method via the optical density measurements in BMG LABTECH GmbH, SpectroStar Nano, Ortenberg, Germany. A concentrated LAB cell was appropriately diluted to 0.05 McFarland standard concentrations (6 Log CFU/mL) at 660 nm. To 100 g of puree, 1 mL of the LAB culture (6 Log CFU/mL) was inoculated and incubated at 37 °C for 2 days; after this, it was stored for 7 days at 4 °C. The non-fermented purees at 0 (*PPD0*) and 2 days (*PPD2*), and stored at 4 °C for 7 days (*PPD7*), were used as controls. Other treatments included papaya puree fermented with *Leu. pseudomesenteroides 56* for 2 days (L56*D2) and stored at 4 °C for 7 days (L56*D7), papaya puree fermented with *W. cibaria 64* for 2 days (W64*D2) and stored at 4 °C for 7 days (W64*D7), and puree fermented with *Lpb. plantarum* 75 for 2 days (L75*D2) and stored at 4 °C for 7 days (L75*D7). Fermented and non-fermented purees were stored at −20 °C prior to analysis, and the fermentation was performed in triplicate.

### 2.4. Physicochemical Properties of Fermented and Non-Fermented Papaya Puree

The physicochemical properties of only pasteurised and fermented papaya puree were determined at 0 and 2 days of fermentation, and 7 days of storage. The pH was measured using the EUTECH pH2700 Instruments (EUTECH Instruments, Illinois, IL, USA), while the total soluble solids (TSS) of samples was measured using the ATAGO PAL-3 pocket refractometer (Atago USA Inc., Tokyo, Japan). The obtained refractive index values were recorded in °Brix. The total titratable acidity of samples was determined according to the method of Reddy et al. (2015). The effect of fermentation and storage on the colour of purees was determined using a CM-3500 d spectrophotometer that made use of spectraMagic NX software (Konica Minolta, Konica Minolta Sensing Inc, Tokyo, Japan). The degree of lightness (L*), red to green component (a*), and yellow to blue (b*) colour components of samples were measured. The calculation of the total colour difference (*∆E*) was performed according to Managa et al. [16].

### 2.5. Determination of Microbial Count and Survival of LABs

The evaluation of the total viable count and surviving LAB count of the puree used pour plating techniques [17]; the plating of the serially diluted samples was on appropriate media. For the total fungal (yeast and mould) counts, the plating of the dilutions was on Yeast Extract Glucose Chloramphenicol Agar (YGCA), bacteria count on nutrient agar (NA) and surviving LAB count on MRS agar plates. The NA plates underwent incubation at 37 °C for 24 h, YGCA plates at 27 °C for 5 days, while the incubation of the MRS plates was performed anaerobically at 30 °C for 48 h. The surviving LAB, aerobic bacterial and fungal counts were enumerated as logarithmic colony forming units per gram (Log CFU/g) of sample.

### 2.6. Organoleptic Properties of Non-Fermented and Fermented Stored Papaya Purees

The sensory evaluation of the puree used a quantitative descriptive analysis technique described by Oliveira et al. [18], with some modifications. The selection of nine trained panellists was from the pool of assessors trained to identify the desired characteristics of the puree. The panellists were composed of healthy male and female research employees. There were two training sections adopted, and the samples were rated using a structured scale ranging from 0 to 6 (absent = 0; 1–2.4 = weak; 2.5–3.9 = moderate; –6 = strong). The assessment of the perception of bright or dark orange colour was performed using ripe papaya juice (100%) and papaya juice with 1% food grade browning as a reference, respectively. The characteristic aroma of papaya was assessed using a ripe papaya pulp juice (100%), while the characteristics of a viscous food in the mouth (consistency) was assessed using (30%) glucose syrup solution as a reference. The assessment of the perception of acid taste and fermented fruit (sourness) was performed by using a commercial unsweetened yoghurt, while the sweet taste characteristics on the tongue were evaluated using sucrose solution (70%) as a reference. A commercial fresh fermented fruit concentrate was used as a reference to determine the overall acceptability. Coded samples were served chilled in white cups with lids to the panellists in a white light-illuminated cubicle. The means of the attributes were calculated, and the cut-off point was set at 2.5 for the acceptability of attributes.

### 2.7. Determination of Total Phenolic Content 

Total phenolic content was determined according to Fessard et al. [3], using 30 µL of 10-fold diluted sample and 150 µL of Folin-Ciocalteu reagent, and afterwards adding 60 µL of 700 mM Na_2_CO_3_ and holding in the dark for 1 h. The absorbance at 760 nm was measured (Infinite M200 PRO, Tecan, Mannedorf, Switzerand) and results expressed in milligrams of gallic acid equivalent (GAE).

### 2.8. Simulated In Vitro Gastrointestinal Digestion

*In vitro* digestion, to test the bioaccessibility of antioxidant compounds, was carried out on ethanolic extract of fruit purees according to Brodkorb et al. [19], mimicking the gastric, intestinal and dialysis phases. A set of 10 g of fruit puree (fermented and non-fermented, stored for 7 days) was mixed with 16 mL of simulated gastric fluid (SGF). The mixture, held at pH 1.3 by adjusting with 6 M HCl, was incubated with freshly prepared pepsin solution (10 mL solution in 0.1 M HCl), sufficient to generate a 142 mg/mL sample. After 2 h, simulated intestinal fluid (SIF) was added to the gastric solution and the pH maintained at 7.0 with 5 M NaOH before adding freshly prepared pancreatin-bile salt solution (39.2 mL of pancreatin + 6 mL of bile salts solution in 1 M NaHCO_3_) to produce a sample of 8.375 mg/mL. The mixture was held for 2 h at 37 °C with shaking at 100 rpm. The collection of the samples (10 mL) was carried out after the intestinal phase, with the remaining used for the dialysis phase. The sample remained at −20 °C to stop intestinal digestion. For dialysis, a dialysis bag (10 cm max, mw cut-off 10 kda) was filled with 5.5 mL NaCl (0.9%) and 5.5 mL NaHCO_3_ (0.5 M) placed in a beaker filled with SIF (70 mL) and incubated for 45 min at 37 °C, with shaking at 100 rpm. For analysis purposes, a 10 mL sample collected from the dialysis bag was lyophilised.

### 2.9. Determination of FRAP Activity

Total antioxidant scavenging activity was determined according to Managa et al. [16] using 0.2 g freeze-dried fruit puree extracted using 2 mL of sodium acetate buffer (pH 3.6). An amount of 220 µL of FRAP reagent solution was placed on a microplate (10 mmol/L TPTZ (2,4,6-tris (2-pyridyl)-1,3,5-triazine) acidified with concentrated HCl and 20 mmol/L FeCl_3_), followed by 15 µL of the homogenised puree extract. The absorbance measurement was performed at 593 nm (Spectrophotometer BMG LABTECH GmbH, SpectroStar Nano, Ortenberg, Germany). The reducing antioxidant power was expressed in Trolox µmol TEAC/100 g DW. 

### 2.10. Effect of Digestion on the Phenolic Profile of Fermented and Non-Fermented Papaya Purees

Extraction and analysis of phenolics in fermented and non-fermented digested purees were performed according to the method of Palafox-Carlos et al. [20]. Digesta from fermented and non-fermented purees were freeze-dried (0.25 g) and homogenised into 10 mL of 80% methanol containing BHT (1 g/L); then, 5 mL of 6 M HCL was homogenised using a BV1000 vortex mixer (Benchmark Scientific Inc., Sayreville, NJ, USA) and the mixture stirred carefully. A 2510 model ultrasonic bath (Branson, LabFriend Pty Ltd., North Sydney, NSW, Australia) sonicated the mixture for 30 min at 70 °C, then centrifuged it at 10,000 rpm for 15 min at 4 °C using a Hermle centrifuge (Model Hermle Z326k, Hermle Labortechnik GmbH, Wehingen, Germany). The collected supernatants were filtered through a 0.22 μm PTFE syringe filter (Grafiltech). The resulting filtrate was injected and analysed in the HPLC/UV-DAD system using a Shimadzu Prominence-i-LC-2030C 3D, Auto Sampler (SIL-20A) HPLC system (Shimadzu, Kyoto, Japan), coupled to a diode array detector for HPLC analysis. Chromatographic separation was achieved using a Shim-pack Gist C18 5 µm, 4.6 × 250 mm reverse phase column with gradient elution at 30 °C, using a 10 µL injection volume. The mobile phase consisted of 6% glacial acetic acid (solvent A) and 75% acetonitrile containing 5% glacial acetic acid (solvent B). The elution gradient was 0–100% (B) for 30 min, kept for 5 min at 100% (B), then returned to 0% (B) for 3 min at a flow rate of 0.6 mL/min. The detection of analytes was performed at 280 nm, and the compounds identified based on a combination of retention time and spectral matching based on standards.

### 2.11. Determination of α-Glucosidase Inhibition 

For the effect of fermentation on the antidiabetic activity of fermented and non-fermented purees, alpha-glucosidase assay was performed using samples obtained on day 7 [21]. Purees were homogenised with sodium phosphate buffer (0.1 M) and dilutions ½, 1/5, 1/10 and 1/20 obtained. An aliquot of 62 µL of sodium phosphate buffer, 50 µL of enzyme and 62 µL of inhibitor sample underwent mixing in a 96-well plate, and incubated for 2 min at 37 °C under shaking. To the wells, 25 μL of substrate solution was added and absorbance measured at 405 nm ((Infinite M200 PRO, Tecan, Mannedorf, Switzerand). The activity and percentage of enzyme inhibition in fruits extracts were determined using Equation (1) and Equation (2), respectively:

OD test_405_ = OD sample − OD negative control (1)

% Glucosidase inhibition = 100% − activity of OD test(2)

### 2.12. Statistical Analysis 

This study used a completely randomised design with five replicates per treatment. The fermentation experiments were performed twice to ensure the reliability of data. One-way analysis of variance (ANOVA) tested the significant differences between the means. Means were compared among the treatments by the least significant difference (LSD) test, at *p* < 0.05, using the Genstat statistical programme for Windows 13th Edition, 2010 (VSN International Hempstead, Hertfordshire, UK). 

## 3. Results and Discussion

### 3.1. Physicochemical Properties of LAB-Fermented Papaya Purees

Lactic acid bacteria have the ability to break down carbohydrates into organic acids [3], which could help in food preservation and enhance the safety of food. The pH of fermented and non-fermented papaya puree was in the range of 3.03 to 5.08 (Table 1). The pH values of purees reduced with fermentation after 2 days compared to the non-fermented sample (5.08). The highest reduction in the pH was obtained in L56*D2 (3.03) and was not significantly different to L75*D2 (3.16) fermented for 2 days, while the non-fermented at day zero had the highest pH (5.08). Moreover, after storage for 7 days, there was a significant increase in the pH of the fermented purees, except the control (*p* ≤ 0.05), relative to values at 2 days of fermentation. The increase in pH after 7 days of storage might have been caused by the poor survival of the LAB due to inappropriate conditions for growth during storage at 4 °C, which could have enabled the growth and release of some yeast metabolites that could raise the pH. The titratable acidity contents of purees ranged from 0.59 to 0.94 mg/mL lactic acid and increased with fermentation relative to the non-fermented sample. The titratable acidity was highest in L75*D2 (0.94 mg/mL) and lowest in the non-fermented puree (0.59 mg/mL) at day zero. The fermented samples were significantly different to the non-fermented samples (*p* ≤ 0.05), while the non-fermented samples at day 0 and 2 were not significantly different to each other (*p* > 0.05). The decrease in the pH with an increase in titratable acidity contents after 2 days of fermentation could be due to the activity of inoculated LABs in converting carbohydrate substrates in the purees into organic acid after two days of fermentation. Ayed et al. [22] reported a similar observation of pH decrease after fermentation in red grape juice. 

Key: *PPD0* = non-fermented papaya puree at 0 days; *PPD2* = non-fermented papaya puree at day 2; L56*D2 = papaya puree fermented with *Leu. pseudomesenteroides* 56 for 2 days; W64*D2 = papaya puree fermented with *W. cibaria 64* for 2 days; L75*D2 = papaya puree fermented with *Lpb. plantarum 75* for 2 d; *PPD7* = non-fermented papaya puree stored at 4 °C for 7 d; L56*D7 = papaya puree fermented with *Leu. pseudomesenteroides 56* stored at 4 °C for 7 d; W64*D7 = papaya puree fermented with *W. cibaria 64* stored at 4 °C for 7 d; L75*D7 = papaya puree fermented with *Lpb. plantarum 75* and stored at 4 °C for 7 d. 

The titratable acidity contents of purees decreased slightly during storage for 7 days, thus signifying a decline in the fermentative activity of the inoculated LABs in the purees due to the possible death of some LAB cells during storage. The increase in pH during storage, as observed in the fermented purees, could be due to a decline in active LAB counts, which corroborates the decrease in puree acidity after storage. The reduced acidity could have enhanced the activities of some competing bacteria and fungi, which utilise organic acids as a carbon source, thereby producing metabolites that lower the pH of the purees [17]. Similar to the reduction in pH observed in this study, LAB fermented emmer -based beverages fortified with fruit juices have been reported to have lower pH after fermentation [23]. The higher degree of acidity characterised by low pH, as observed in L75*D2 at day 2 of fermentation and 7 days of storage, is suggestive of *Lpb. plantarum* being a strong hetero-fermenter that could survive at low pH and temperature.

The TSS content of the fermented and non-fermented purees ranged from 6.83 to 8.60 °Brix (Table 1) and was highest in L56*D7 and lowest in L75*D2. The TSS content was significantly reduced after fermentation for 2 days compared to the non-fermented sample (*p* ≤ 0.05). However, L56*D2 and L75*D2 were not significantly different from each other, in line with the control at day 0 and 2 (*p* > 0.05). The reduction in the TSS contents of the fermented purees compared to the non-fermented purees could be due to the metabolic activities of inoculated LAB cultures that break down, reducing sugars into organic acid, confirmed by a reduction in the pH of the purees. The reduction in the TSS content after fermentation is in agreement with that obtained by Soibam et al. [24] in fermented sugarcane and beet juice. The TSS contents of purees increased during storage for 7 days. Samples L56*D7 (8.60 °Brix) and non-fermented (8.50 °Brix) puree were not significantly different (*p* > 0.05) from each other after 7 days of storage, in line with samples L75*D7 and W64*D2 (7.13 °Brix). The increase in the TSS content during storage could be due to the hydrolysis of carbohydrates into reducing sugars, thus supporting the previous reports of an increase in the total soluble solid of stored kinnow juice [25] and carrot–orange juice [26].

### 3.2. Effect of Fermentation and Storage on Colour Characteristics of Papaya Puree

The effect of fermentation and storage on the colour characteristics of papaya puree is presented in Table 2. The luminosity of fermented and non-fermented puree ranged from 29.96 to 43.21 and was highest in the non-fermented puree prior to fermentation, and lowest in L75*D7 papaya puree stored for 7 days. After fermentation and storage at cold temperature (4 °C), the luminosity (L*) of the puree reduced relative to the control. The purees fermented for 2 days were significantly different to the non-fermented purees at day 2 (*p* ≤ 0.05). The L56*D2 and W64*D2 were not significantly different (*p* > 0.05), while L75*D2 significantly differed to others in terms of luminosity (*p* ≤ 0.05). The type of fermenting LAB cultures significantly influenced the colour parameters of the puree. Contrary to that obtained in other treatments, W64*D7 had increased lightness upon storage for 7 days, thus suggesting the potential ability of *W. cibaria 64* to inhibit enzymatic oxidation in samples.

The redness to greenness characteristics (a*) of the puree ranged from 23.15 to 26.38. The degree of redness to greenness of the puree did not significantly increase with fermentation and storage and was highest in L75*D7 (26.38). However, a* in L56*D2 and W64*D7 increased after fermentation and storage for 7 days, respectively, while L56*D2 was not significantly different to the control before fermentation (*p* > 0.05). The yellow to blue components of the puree was in the range 50.61–58.02 and was highest in the non-fermented sample. At day 2 of fermentation, the b* component of fermented samples was significantly different to the control, except W64*D2, while upon storage, samples were not significantly different to the control, except L75*D7. The decreased luminosity of the puree and high colour change in stored fermented papaya puree could be due to an enzymatic oxidation caused by the reduced fermentation and acidity in the puree during storage. The b* colour coordinate related to the yellow colour of the puree used for the calculation of *ΔE* relates to the colour change. 

The *ΔE* relates to the colour change, and is associated with the rate of enzymatic browning in fruit juices and purees [26]. The *ΔE* of the samples ranged from 9.8 to 13.8. The colour change in the puree was lowest in L56*D2 and highest in L75*D7. The *ΔE* significantly increased by fermentation and storage (*p* ≤ 0.05); therefore, fermenting with different types of LAB strains and storage at a cold temperature influenced the *ΔE* in papaya puree. There was a significant colour change in papaya puree, as its *ΔE* values were greater than two. The colour change in fruit and juices correlate with the enzymatic activities of polyphenolic oxidase [27]. Hence, the high *ΔE* values in stored fermented purees could be due to an auto-oxidation of polyphenolic compounds [28]. 

Key: *PPD0* = non-fermented papaya puree at 0 days; *PPD2* = non-fermented papaya puree at 2 d; L56*D2 = papaya puree fermented with *Leu. pseudomesenteroides 56* for 2 d; W64*D2 = papaya puree fermented with *W. cibaria 64* for 2 d; L75*D2 = papaya puree fermented with *Lpb. plantarum 75* for 2 d; *PPD7* = non-fermented papaya puree stored at 4 °C for 7 d; L56*D7 = papaya puree fermented with *Leu. pseudomesenteroides 56* stored at 4 °C for 7 d; W64*D7 = papaya puree fermented with *W.cibaria 64* stored at 4 °C for 7 d; L75*D7 = papaya puree fermented with *Lpb. plantarum 75* and stored at 4 °C for 7 d.

### 3.3. Survival of LABs in Papaya Purees after Fermentation and Storage

**Figure 1:**
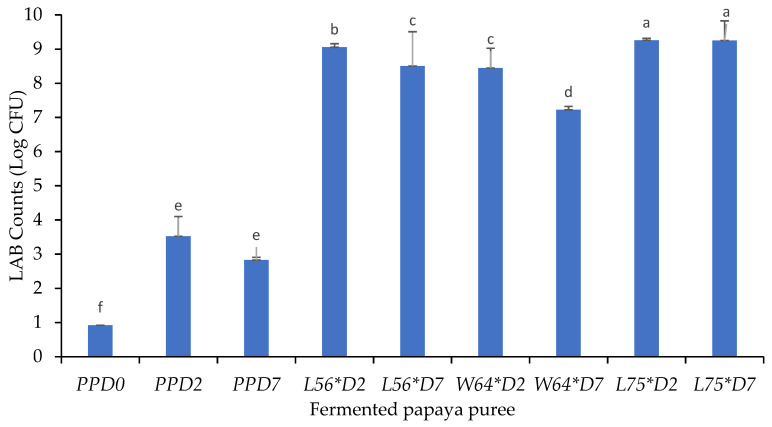
Surviving lactic acid bacteria counts in papaya puree. Bar with different letters are significantly different are significantly different at *p* < 0.05.

The surviving LAB counts in fermented and non-fermented purees ranged from 0.92 to 9.25 Log CFU/g in puree (Figure 1). As expected, there was a significant increase in the LAB count of purees fermented for 2 days, while upon storage for 7 days, there was a significant decrease, except in L75*D7, which was more stable after storage (*p* ≤ 0.05). Samples L75*D2 (9.25 Log CFU/g) and L75*D7 (9.24 Log CFU/g) were significantly higher than other fermented and non-fermented samples and were not significantly different from one another (*p* ≤ 0.05). This suggests the stability and survival of *Lpb. plantarum*-fermented papaya purees at acidic condition after storage for 7 days. *Lpb. plantarum* prefers glucose and lactose as a carbon source and can easily adapt to different conditions [29], which accounts for its versatile use in fermentation. The stability of L75*D7 puree suggests its potential to be used as functional food (probiotics) that can help to manage dysbiosis in the gastrointestinal tract. *Lpb. plantarum* produces antimicrobial compounds, such as plantaricin, which can inhibit the growth of spoilage and pathogenic microorganisms [30]. At day 2 of fermentation, fermented purees were significantly different from others and the non-fermented samples; however, after storage, W64*D2 was not significantly different to L56*D7. The surviving LAB counts significantly decreased after storage, except in L75*D7; this might be associated with the inactivation or death of some LAB cultures due to unfavourable growing conditions during storage. The survival of *Lpb. plantarum* in the puree after storage supports the finding of Srisukchayakul et al. [31] on the acid tolerance of *Lpb. plantarum* in fruit juices stored in refrigerated conditions. The highest LAB count decrease in fermented purees was in W64*D7. Thus, *W. cibaria 64* culture might not be able to survive cold storage (4 °C) for 7 days unlike the *Lpb. plantarum*-fermented papaya puree. The variation in the LAB cell survival of fermented mango puree after storage could be due to the unique characteristics of individual LAB cultures used in the study. The fungal counts (1–3 Log CFU/g) and total viable bacteria (data not shown) were within the acceptable limits for fruit juices [32].

Values are the mean ± standard deviation, and means followed by a different letter within the row are significantly different (*p* ≤ 0.05). Key: *PPD0*: non-fermented papaya puree at 0 days; *PPD2*: non-fermented papaya puree at day 2; L56*D2: papaya puree fermented with *Leu. pseudomesenteroides* 56 for 2 days; W64*D2: papaya puree fermented with *W. cibaria* 64 for 2 days; L75*D: papaya puree fermented with *Lpb. plantarum* 75 for 2 days; *PPD7*: non-fermented papaya puree stored at 4 °C for 7 d; L56*D7: papaya puree fermented with *Leu. pseudomesenteroides* 56 stored at 4 °C for 7 d; W64*D7: papaya puree fermented with *W. cibaria* 64 stored at 4 °C for 7 d; L75*D7: papaya puree fermented with *Lpb. plantarum* 75 and stored at 4 °C for 7 d; CFU/g: colony forming units per gram of samples.

### 3.4. Organoleptic Properties of Fermented and Non-Fermented Papaya Puree

**Figure 2:**
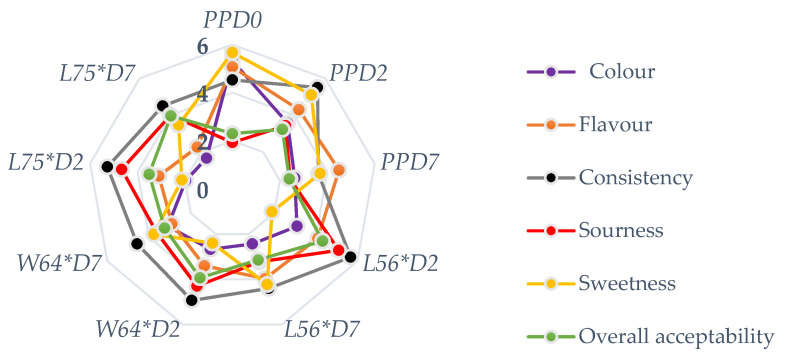
Sensory attributes of pasteurised and fermented papaya puree.

The sensory properties of fermented and non-fermented papaya puree are presented in Figure 2. The colour perception ranged from a dull orange (1.67) to a strong bright orange colour (5.33) and was highest in the non-fermented samples at day zero. Similarly, the aroma attributes ranged from weak aroma (2.27) to strong aroma (5.07) perception and was highest in the non-fermented samples at day zero. The perception of the bright orange colour of papaya significantly decreased with storage and was influenced by the type of LAB fermenting the purees (*p* ≤ 0.05). After storage, sample L56*D7 was not significantly different to the non-fermented puree at day 7 in terms of colour and aroma, while a contrary perception was made in W64*D7 and L75*D7 (*p* > 0.05). The decrease in lightness could be due to browning caused by an auto-oxidation of the poly-phenolic compounds in the samples due to possible exposure to metal ions [28]. The lower rating in flavour, especially in *Lpb. plantarum* fermented samples, could be due to the fact that the cultures are known as fermentative heterolactic microorgansims that exclusively produce lactic acids [33], unlike *Leu. pseudomesenteroides* and *W. cibaria,* which are able to produce flavour compounds aside from organic acids during fermentation. 

The consistency scores of the product ranged from moderate (3.63) to strong (5.67) and were highest in L56*D2 and lowest in *PPD7* puree stored for 7 days. The sourness describes the acid taste of the samples, and its perception ranged from a weak (1.93) to strong (5.10) acid taste. The perception of sour taste increased with fermentation but decreased with storage, thus supporting the lower pH in samples after fermentation and the upward increase in pH during storage. The sourness was highest in L56*D2; however, it was not significantly different to L75*D2 and W64*D2 after fermentation (*p* > 0.05), but differed to the control (*PPD0*, *PPD2*, *PPD7*). Sweetness is the perception of a sweet taste, and it is desirable in some foods, such as purees or juices. The sweetness ranged from weak (1.90) to strong (5.67) sucrose perception and was highest in the non-fermented samples at the start of the experiment (*PPD0*). The sweetness decreased with fermentation and increased during storage. This agrees with the observation made in this study on the decrease in TSS during fermentation and its slight increase after storage for 7 days. The increase in the sweet taste could have been a product of the de-polymerisation of polysaccharides or other complex carbohydrates in the papaya puree during storage, thus supporting the assertion that slight fermentation could proceed during cold storage, as earlier reported by Managa et al. [34]. The overall acceptability of the fermented and non-fermented stored puree was in the range of 2.30–4.33 (weak to strong) in the product. The highest acceptability was obtained in L56*D2 but was not significantly different to L75*D7 (3.97) stored for 7 days, which was slightly different to L56*D7 and W64*D7. Hence, stored puree fermented with *Lpb. plantarum* could deliver a probiotic and nutrient-dense and acceptable product.

Key: *PPD0* = non-fermented papaya puree at 0 days; *PPD2* = non-fermented papaya puree at day 2; L56*D2 = papaya puree fermented with *Leu. pseudomesenteroides* 56 for 2 days; W64*D2 = papaya puree fermented with *W. cibaria 64* for 2 days; L75*D2 = papaya puree fermented with *Lpb. plantarum 75* for 2 days; *PPD7* = non-fermented papaya puree stored at 4 °C for 7 d; L56*D7 = papaya puree fermented with *Leu. pseudomesenteroides 56* stored at 4 °C for 7 d; W64*D7 = papaya puree fermented with *W. cibaria 64* stored at 4 °C for 7 d; L75*D7 = papaya puree fermented with *Lpb. plantarum 75* and stored at 4 °C for 7 d.

### 3.5. Changes in Phenolic Compounds and Antioxidant Power in Papaya Puree after Fermentation and Storage

The total phenol content and antioxidant capacity and concentration of different phenolic compounds in fresh papaya puree at day 0, non-fermented and LAB fermented papaya puree stored for 7 d at 4 °C are shown in Table 3. 

The non-fermented papaya puree at 0 d contained the lowest TPC (303.9 mg/100 g DW), while L75*D7 was the highest (475.1 mg/100 g DW) compared to the non-fermented and fermented purees stored at 4 °C for 7 d. The L56*D7 was the lowest with regard to total phenol content (451.0 mg/100 g DW) among the other fermented purees (W64*D7 and L75*D7). There was an increase in the total phenol content of lactic acid in fermented plant-based food [35]. Lactic acid bacteria, such as *Lpb. plantarum*, reportedly hold the ability to remove sugar moieties and hydrolysed galloyl moieties from phenolic compounds during fermentation [36]. The higher total phenol content in *Lpb. plantarum*-fermented papaya puree corroborates the report in *Lpb. plantarum*-fermented blueberry juice [37] and could be attributable to both the hydrolysis of glucosides to aglycones and possibly the production of esterases to which hydrolyses glycosides ester bonds, which could aid the release of insoluble bound and conjugated phenolic compounds [38] in the purees. 

The *Leu. pseudomesenteroides 56* could have participated in the partial conversion of simple phenolic compounds and depolymerisation of large molecular weight phenols in papaya puree [38]. To avoid the overestimation of the total phenol content through the spectrophotometric method, there were investigations performed on the changes in individual phenolic compounds during fermentation. The non-fermented puree at 0 d, and fermented papaya puree stored at 4 °C for 7 d contained gallocatechin gallate, gallic acid, protocatechuic acid, vanillic acid, syringic acid, ellagic acid, chlorogenic acid, catechin, epicatechin, quercetin, p-coumaric acid and ferulic acid. Gallocatechin gallate was the most abundant phenolic compound in the non-fermented puree at 0 (581.4 mg/kg) and 7 days of storage (564.2 mg/kg). Gallocatechin gallate reduced significantly (*p* < 0.05) during the fermentation and storage of purees at 4 °C for 7 d. L56*D7 puree had a 62.34% reduction in gallocatechin gallate compared to the non-fermented puree (*PPD0*). Contrary to that observed in gallocatechin gallate, an increasing trend was observed with catechin after fermentation, with L56*D7 having a significantly (*p* ≤ 0.05) higher concentration (66.1 mg/kg). However, the observation suggests that *Leu. pseudomesenteroides 56* could biotransform gallocatechin gallate to corresponding catechins by its esterase enzymes. The stability of catechins is pH dependent, and they are stable in acidic solution during lactic acid fermentation due to the production of lactic acid [39]; hence, the higher concentration of catechin in L56*D2 could be due to its high acidic condition, thus resulting in the stabilisation of catechin and epicatechins. Furthermore, the disintegration of the cell wall could have further favoured the extraction of the catechins into the puree [40]. Sample L56*D7 had the highest protocatechuic acid, p-coumaric acid and ferulic acid concentrations compared to the other fermented and non-fermented purees at 0 and 7 days. The increase in the phenolic acids in the other LAB-fermented purees could be due to the mobilisation of the bound phenolics to a free state via the enzymatic hydrolysis that occurs during fermentation, which could increase their bioavailability [41]. Additionally, L56*D7 and L75*D7 showed a significant (*p* < 0.05) increase in vanillic and ellagic acid, respectively. The hydrolysis of ellagitannins during fermentation could have freed and increased the ellagic acid content [42]. Rodríguez et al. [43] reported the ability of LABs to decarboxylate and convert p-coumaric acids to their corresponding vinyl derivatives, which could account for the decrease in p-coumaric acid concentrations in W64*D7 and L75*D7. Contrary to a four-fold increase in quercetin during fermentation initiated by *Lpb. plantarum C2* in Myrtle berries [44], quercetin concentration declined during fermentation in papaya puree. Therefore, the changes in the concentration of phenolic compounds during biotrasformation and metabolic activity depend on the type of LAB strain, and the enzyme systems involved in fermentation, nutrient composition and intrinsic factors of fruit [45].

### 3.6. Effect of LAB-Fermented Papaya Puree on In Vitro α-Glucosidase Inhibition Activity

**Figure 3:**
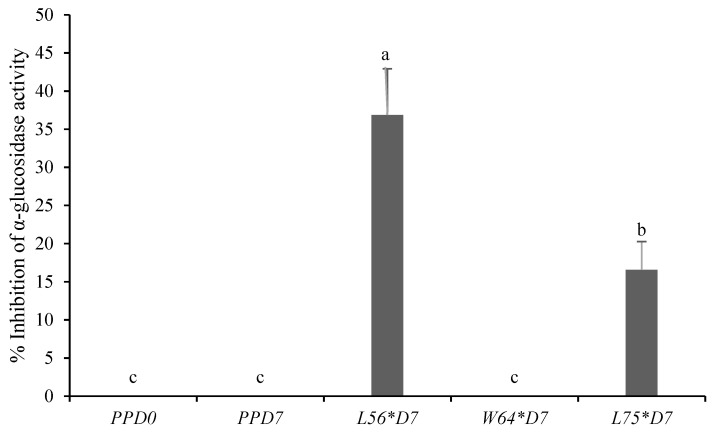
Inhibition of α-glucosidase activity of ½ diluted papaya puree fermented with different LAB strains. Bars with the same letter are not significantly different at *p* < 0.05.

The α-glucosidase inhibitory activity of stored fermented and non-fermented papaya purees is presented in Figure 3. The percentage α-glucosidase inhibitory activity ranged from 0% to 37%, of which L56*D7 (37%) was significantly (*p* < 0.05) higher than L75*D7 (17%), while other samples had no α-glucosidase inhibitory activity and were comparable to the non-fermented purees at 0 and 7 days. The higher concentrations of protocatechuic acid, catechin, epicatechin, caffeic acid, p-coumaric acid and ferulic acid in L56*D7 could have contributed towards the inhibition of α-glucosidase activity. Protocatechuic acid (r = 0.99, *p* < 0.05), catechin (r = 0.99, *p* < 0.05), epicatechin (r = 0.64 *p* < 0.05) caffeic acid (r = 0.77 *p* < 0.05), p-coumaric acid (r = 0.94, *p* < 0.05) and ferulic acid (r = 0.94, *p* < 0.05) showed a strong positive correlation with the α-glucosidase inhibitory activity. A significant positive correlation was established between FRAP activity and α-glucosidase (r = 0. 0.88, *p* < 0.05). Phenolic compounds inhibited the intestinal α-glucosidase activity, and are regarded as a mechanism to exert antidiabetic effects [46]. α-glucosidase facilitates glucose absorption in the intestines; thus, inhibiting this enzyme could help to reduce the glucose absorption rate and alleviate postprandial hyperglycaemic condition [47].

Data are presented as the mean and standard deviation. Bars with different letters indicate significant differences at *p* ≤ 0.05. *PPD0*: papaya puree at 0 days of storage; *PPD7*: non-fermented puree stored at 4 °C for 7 days; L56*D7: papaya puree fermented with *Leu. pseudomesenteroides* 56 stored at 4 °C for 7 days; W64*D7: papaya puree fermented with *W. cibaria* 64 stored at 4 °C for 7 d; L75*D7: papaya puree fermented with *Lpb. plantarum* 75 and stored at 4 °C for 7 d.

### 3.7. In Vitro-Simulated Gastrointestinal (GI) Digestion and Antioxidant Power of Fermented and Non-Fermented Papaya Puree 

In order for consumers to be able to utilise the phenolic compounds in food, the increase in the bioaccessibility of polyphenols is important [48]. Therefore, the effect of digestion on the phenolic components, percent recovery and bioaccessibility of fermented and non-fermented purees at the gastric, intestinal and dialysis phase is presented in Table 4 and Table 5, respectively. 

At the gastric phase, the total phenol content was significantly higher (502.4 mg/100 g DW) in stored L75*D7 papaya puree. In general, gastric, intestinal and dialysable fractions of non-fermented and fermented puree showed significantly (*p* < 0.05) higher concentrations of phenolic content compared to the respective undigested sample (before fermentation). Therefore, the observed differences could relate to the interaction and interference of the food matrix and interactions with other dietary components, such as fibre, proteins, pH and the enzyme pancreatin. This observation could be due to the hydrolysis of bound phenolic compounds from carbohydrates and proteins from the food matrix facilitated by enzymatic action and low pH [49]. The decrease in pH during fermentation could have increased their stability and extractability [50]. 

Conversely, the observed differences in total phenol content in the gastric fraction could be due to the difference in the survival or cell population of the LAB strains in the gastrointestinal phase, which is responsible for the higher metabolism and biotransformation of most phenolic compounds [8]. However, the cell population of the LAB strains in the gastrointestinal phase were not quantified in this study. The reduction in the phenolic content in the intestinal fraction was related to the pH changes from acidic to alkaline pH [51]. Furthermore, the molecular arrangement of the different bioactive molecules or interaction effects between the bioactive compounds and other dietary compounds could have affected the total phenolic content in the dialysed fraction [9]. 

The undigested non-fermented puree stored for 7 days at 4 °C contained the highest concentration of gallocatechin gallate (564.2 mg/kg), and its concentration significantly (*p* < 0.05) decreased with fermentation, while W64*D7-fermented puree had the highest concentration (462.5 mg/kg). The gallocatechin gallate concentration at the intestinal phase of all fermented and non-fermented purees showed a substantial reduction, varying from 264.3 to 100.4 mg/kg compared to the gastric fraction and the undigested purees. The % recovery of gallocatechin gallate in the intestinal fraction was 46.8%, 45.9%, 34.3% and 33.7% in *PPD7*, L56*D7, W64*D7 and L75*D7, respectively, while 14.5% was recovered in the L75*D7 dialysed fraction. The intestinal fractions of non-fermented and fermented purees showed a substantial increase in gallic acid concentration compared to the undigested and gastric fractions. The amount of gallic acid bioaccessible in the dialysable fraction of the non-fermented puree was 1100.0% compared to its undigested sample. The % recovery of gallic acid in the dialysed fractions of W64*D7, L56*D7 and L75*D7 was 1479.3%, 850% and 675.0%, respectively.

Krook and Hagerman [51] reported the stability of epigallocatechin-O-gallate at pH < 1.5 and 5–6, and its degradation at pH higher than seven produced gallic acid. Therefore, it can be hypothesised that the gallocatechin gallates could be stable at pH 2, and at pH 7 due to its instability, and could undergo decomposition that produces gallic acid, especially at the intestinal phase. Furthermore, Liu et al. [52] also showed that the increase in gallic acid due to alkaline hydrolysis could had released the bound phenolic acids, thus increasing their bioavailability. On the contrary, Tagliazucchi et al. [52] and Jara-Palacios et al. [53] reported the degradation of gallic acid at the intestinal phase. However, the L56*D7 and L75*D7 could have partially metabolised, thereby reducing the % recovery of gallic acid in the intestinal fraction of the fermented purees. 

Similar to the report of Jara-Palacios et al. [53] on the higher concentration of protocatechuic acid in the intestinal digests of Zalema grapes (*Vitis vinifera* sp.) pomace. Lui et al. [54] also showed increased extraction of phenolic acids under mild alkaline conditions.

In this study, samples W64*D7 and L75*D7 had higher protocatechuic acid concentrations than the undigested samples (PPD7). Additionally, the higher gallic acid concentration at the intestinal phase indicates that the gallic acid did not undergo a dehydroxylation process for the production of protocatechuic acid [9]. There was a significantly higher concentration of protocatechuic acid in the W64*D7 and L75*D7 at the gastric phase (*p* < 0.05) than the L56*D7. The % recovery of protocatechuic acid was significantly higher in the W64*D7- (342.5%) and L75*D7 (325.3%)-fermented purees at the intestinal phase compared to the L56*D7 and undigested purees (*p* ≤ 0.05). W64*D7 and L75*D7 had a significantly higher % recovery of protocatechuic acid at the dialysis phase compared to the other samples. It is possible that the protocatechuic acid could have been partially metabolised by L56*D7 during gastric digestion, since it was reduced from 44.4 to 32.1 mg/kg. 

Catechin concentration increased in the gastric fraction of the fermented puree. This could be due to the lower pH of the fermented purees and the lower gastric pH, which resulted in stable catechin molecules [52]. At the same time, catechin concentration increased significantly in the intestinal fractions of fermented purees. The intestinal fraction of L75*D7 showed a significantly higher amount (277.5 mg/kg) of catechins with 536.8% recovery. Moreover, the dialysable fraction of L75*D7 showed the highest bioaccessible catechin (132.1%) compared to the undigested, digested fermented and non-fermented purees. The stability of catechins has been correlated with the pH and are reported to be stable in acidic conditions, and unstable at pH greater than or near neutral [55]. The observed increase in catechin in the intestinal fraction in this study could be due to the spontaneous degradation of gallocatechins at alkaline pH [9]. 

The percentage recovery of ellagic acid in the gastric fraction of L56*D7 (69.4%) was lower when compared to its undigested sample (103.7%), W64*D7 (154.8%) and L75*D7 (155.6%). This suggests the possible utilisation of ellagic acid by L56*D7. Conversely, the % of ellagic acid was significantly (*p* < 0.05) increased in the intestinal fractions of the non-fermented and fermented puree compared to their undigested samples. The observed increase in ellagic acid concentration could be due to the hydrolysis of ellagitannins from the food matrix to ellagic acid due to the mild alkaline pH (7.5) at the intestinal phase [56]. The highest % bioaccessibility of ellagic acid was in the W64*D7 (200.0%) dialysed fraction and was significantly different to the dialysed digest of the other purees. The observed lower % bioaccessibility (22.6%) of ellagic acid in the dialysed fraction of L75*D7 digest could be due to the possible utilisation of ellagic acid during fermentation caused by *Lpb. plantarum*. *Lactobacillus* spp. reportedly has the ability to utilise ellagic acid and glycosyl ellagic acid during metabolism [57].

Furthermore, the percentage recovery of chlorogenic acid and syringic acid was significantly higher in the intestinal fraction of W64*D7 (769.2%; 772.0%)*,* with the highest % bioaccessible amount of 369.2% chlorogenic acid and 136.0% syringic acid at the dialysis phase. Moreover, fermentation increased the % recovery of vanillic acid in the intestinal fractions and was highest in L75*D7 (508.0%). The highest percentage bioaccessible content of 100% was obtained in W64*D7 and L75*D7 in the dialysable fractions of fermented purees. The increase in ellagic, chlorogenic, syringic and vanillic acid concentrations after intestinal digestion could be due to their release from their bound form in the food matrix due to enzymatic digestion [9]. The decrease in chlorogenic acid, ellagic acid and ferulic acid in L56*D7 at the gastric phase could be due to the partial use of these compounds during metabolism. 

LAB fermentation caused an increase in the quercetin content of fermented purees compared to the non-fermented purees. The percentage recovery of quercetin content was higher in the W64*D7 (119.7%; 110.0%) and L75*D7 (127.4%; 119.4%) compared to L56*D7 (71.9%; 83.2%) and *PPD7* (60.5%; 84.3%) at both intestinal and gastric phases, respectively. L75*D7 had the highest % recovery of quercetin at the intestinal phase and was not significantly different to W64*D7 (*p* > 0.05). Likewise, the dialysable fractions of L56*D7 (41.7%) and W64*D7, despite being high, were not significantly different to one another (*p* > 0.05). A similar non-significant change in the quercetin content was reported during the gastric and intestinal phase digestion of onions [58]. These results, therefore, suggest that the pH change during fermentation and during the gastric, intestinal phases and the action of digestive enzymes (pancreatin), could have participated in the release of quercetin from the food matrix [58]. The degree of metabolization by different LAB strains used in this study varied, as reflected in the p-coumaric acid concentration found in the fermented samples. The observed non-significant reduction in p-coumaric acid in the undigested L56*D7 puree could be due to the decarboxylation of ferulic acid [59]. 

The intestinal and dialysed fractions of W64*D7 had the highest p-coumaric acid % recovery (504.0%) and % bioaccessibility (138.7%) and were significantly higher than those of other purees. A possible reason for the higher recovery of ferulic acid at the dialysis phase, with respect to the undigested samples, could be due to an interference from the food matrix and the reduced esterification of ferulic acid with sugar moieties after digestion [60]. Similarly, a higher % recovery of ferulic acid was observed in W64*D7 and L75*D7 intestinal fractions than their respective gastric fractions. L75*D7 had the highest % bioaccessible ferulic acid at the dialysable fraction. The observed decrease in the % recovery of different phenolic constituents at the intestinal phase in L56*D7 suggests a partial metabolism of the phenolic compound. Valero-Cases et al. [8] previously reported the impact of LAB fermentation on the *in vitro* digestion and biotransformation of phenolic compounds in fermented pomegranate juices. Therefore, the relationship among the concentrations of phenolic compounds in fermented puree could be correlated with the increased LAB survival in L75*D7 and L56*D7 during and after fermentation.

### 3.8. Effect of Fermentation and In Vitro Digestion on the Antioxidant Capacity of Papaya Puree

The types of transformations, such as epimerisation, degradation, oxidation and hydrolysis, during the fermentation and gastrointestinal digestion can affect the phenolic content and its structure [8]. Fermentation with LAB strains increased the antioxidant capacity (FRAP values) of papaya purees, with the exception of L56*D7. During gastric digestion, the FRAP values of *PPD7*, W65*D7 and L75*D7 significantly (*p* ≤ 0.05) increased, compared to the undigested samples. The highest FRAP antioxidant power was obtained in the gastric fraction of L75*D7 (4.7 µmol TEAC/100 g FW), while a significant (*p* ≤ 0.05) decrease in FRAP was observed in the intestinal fractions of L56*D7 and L75*D7, but not W64*D7, when compared to the gastric fractions. The FRAP values in the dialysable fractions range was lowest in *PPD7* (1.1 µmol TEAC/100 g FW) and highest in L75*D7 (2.3 µmol TEAC/100 g FW). The findings in this study confirm that fermentation with *Lpb. plantarum 75* and *W. cibaria 64* increases the FRAP activity due to the contribution of the free soluble antioxidants. The FRAP activity and total phenol content are positively correlated [46]. The metal chelating properties of phenolic constituents contributed to the antioxidant activity of W64*D7 and L75*D7, which can be justified by the increase in most phenolic constituents compared to in the L56*D7 and non-fermented papaya puree.

## 4. Conclusions 

The results presented in this study showed that the fermentation of papaya puree by *W. cibaria 64* and *Lpb. plantarum 75* improves antioxidant capacity (FRAP activity) due to the increase in phenolic constituents compared to the *Leu. pseudomesenteroides 56* and non-fermented papaya puree. However, the viability of all LAB strains used in this study after *in vitro* digestion requires investigation. This study provided important information on the estimation based on the percentage recovery of different phenolic constituents that are available for *in vivo* absorption after the consumption of LAB-fermented papaya puree. However, further investigations are necessary in the survival of LABs; antioxidant activity after in vitro digestion and the bioaccessibility of phenolic constituents after digestion could be investigated using Caco-2 cellular models to confirm the uptake of phenolic and carotenoid components. Based on the phenolics profiles, antioxidants, LAB survival and quality parameters of the purees, the study recommends that local food manufacturers in Reunion Island use *Lpb. plantarum 75* for the fermentation of papaya purees for optimum nutrient bioaccessiblity and functional benefits from locally produced papaya. 

## Figures and Tables

**Table 1:** Changes in pH and total soluble solids (°Brix) of non-fermented and fermented papaya puree.

Fruit Puree	pH	Titratable Acidity (mg/mL)	Total Soluble Solids (Brix°)
*PPD0*	5.08 ± 0.01 ^a^	0.59 ± 0.04 ^d^	8.03 ± 0.06 ^a,b^
*PPD2*	4.99 ± 0.01 ^a,b^	0.63 ± 0.02 ^d^	8.07 ± 0.06 ^a,b^
*PPD7*	4.36 ± 0.01 ^b^	0.68 ± 0.02 ^c^	8.50 ± 0.02 ^a^
L56*D2	3.03 ± 0.01 ^d^	0.76 ± 0.02 ^b^	6.87 ± 0.06 ^d^
L56*D7	3.88 ± 0.01 ^c^	0.73 ± 0.02 ^b^	8.60 ± 0.02 ^a^
W64*D2	3.76 ± 0.01 ^c^	0.80 ± 0.02 ^a,b^	7.13 ± 0.06 ^c^
W64*D7	4.09 ± 0.01 ^b^	0.71 ± 0.02 ^b^	7.80 ± 0.17 ^b^
L75*D2	3.16 ± 0.01 ^d^	0.94 ± 0.04 ^a^	6.83 ± 0.06 ^d^
L75*D7	3.36 ± 0.01 ^c^	0.84 ± 0.04 ^a,b^	7.13 ± 0.07 ^c^

Values are mean± standard error of means; means followed by a different letter within the column are significantly different (*p* ≤ 0.05).

**Table 2:** Colour characteristics of fermented and non-fermented papaya puree.

Fruit Puree	L*	a*	b*	*ΔE*
*PPD0*	43.21 ± 0.01 ^a^	23.15 ± 0.01 ^c^	52.76 ± 0.01 ^b,c^	
*PPD2*	35.07 ± 0.06 ^b^	24.48 ± 0.01 ^b^	58.02 ± 0.01 ^a^	9.8 ± 0.5 ^g^
*PPD7*	32.43 ± 0.01 ^d^	25.62 ± 0.01 ^a,b^	54.42 ± 0.01 ^b^	11.2 ± 1.0 ^d^
L56*D2	33.91 ± 0.08 ^c^	23.87 ± 0.03 ^c^	52.33 ± 0.01 ^c^	9.3 ± 0.1 ^h^
L56*D7	32.13 ± 0.01 ^d^	25.33 ± 0.01 ^a,b^	53.86 ± 0.01 ^b^	11.3 ± 1.0 ^c^
W64*D2	32.99 ± 0.02 ^c^	24.97 ± 0.02 ^b^	53.13 ± 0.15 ^b,c^	10.4 ± 0.1 ^e^
W64*D7	33.65 ± 0.01 ^c^	24.35 ± 0.02 ^b^	55.72 ± 0.01 ^a,b^	10.1 ± 0.7 ^f^
L75*D2	31.29 ± 0.01 ^d,e^	24.97 ± 0.02 ^b^	50.62 ± 0.01 ^d^	12.3 ± 2.3 ^b^
L75*D7	29.96 ± 0.05 ^e^	26.38 ± 0.02 ^a^	50.61 ± 0.01 ^d^	13.8 ± 0.4 ^a^

Values are mean± standard error of means; means followed by a different letter within the column are significantly different (*p* ≤ 0.05). L* = degree of lightness; a* = red to green component; b* = yellow to blue; *∆E* = total colour difference.

**Table 3:** Changes in total phenols, antioxidant capacity and phenolic compounds of stored non-fermented and fermented papaya purees.

Parameters	*PPD0*	*PPD7*	L56*D7	W64*D7	L75*D7
Total phenol (mg GAE/100 g FW)	303.9 ± 0.7 ^e^	408.7 ± 0.8 ^d^	451.0 ± 0.6 ^c^	467.1 ± 0.5 ^b^	475.1 ± 1.9 ^a^
FRAP (µmol TEAC/100 g FW)	1.4 ± 0.2 ^c^	2.0 ± 0.3 ^b^	2.0 ± 0.3 ^b^	2.7 ± 0.5 ^a^	2.8 ± 0.2 ^a^
Phenolic compounds (mg/kg)					
Gallic acid	4.4 ± 1.0	6.7± 1.5	5.6 ± 0.1	2.9 ± 0.2	6.4 ± 0.5
Gallocatechin gallate	581.4 ± 2.7 ^a^	564.2 ± 1.8 ^a^	218.9 ± 2.4 ^d^	462.5 ± 9.8 ^b^	330.4 ± 11.0 ^b^
Protocatechuic acid	19.4 ± 0.4 ^b^	19.3± 0.6 ^b^	44.4 ± 2.0 ^a^	19.3 ± 0.8 ^b^	17.4 ± 0.1 ^b^
Catechin	14.21 ± 2.3 ^c^	14.7 ± 1.5 ^c^	66.1 ± 2.7 ^a^	58.2 ± 0.7 ^b^	51.7 ± 1.3 ^b^
Epicatechin	7.7 ± 0.9 ^b^	6.2 ± 1.2 ^b,c^	16.9 ± 0.7 ^a^	7.7 ± 1.1 ^b^	5.4 ± 0.4 ^c^
Chlorogenic acid	19.9 ± 0.1 ^a,b^	17.2± 0.4 ^b^	17.7 ± 0.2 ^b^	1.3 ± 0.8 ^c^	1.7 ± 0.2 ^c^
Vanillic acid	5.6 ± 0.6 ^a^	4.5± 0.8 ^a^	4.5 ± 0.4 ^a^	2.5 ± 0.5 ^b^	2.5 ± 1.1 ^b^
Syringic acid	4.3 ± 0.1	4.9± 0.1	7.2 ± 0.4	2.5 ± 0.1 ^e,f^	4.8 ± 1.2 **
Ellagic acid	3.9 ± 2.0 ^c^	5.4± 1.1 ^b^	6.2 ± 1.5 ^b^	3.1 ± 0.2 ^c^	12.4 ± 0.2 ^a^
Quercetin	104.9 ± 0.1 ^a^	103.8± 16.3 ^a^	38.1 ± 1.5 ^c^	52.8 ± 5.0 ^b^	57.7 ± 2.3 ^b^
p-Coumaric acid	26.9 ± 0.2 ^b^	23.7± 2.5 ^b^	37.4 ± 3.2 ^a^	17.3 ± 0.8 ^c^	25.4 ± 0.8 ^b^
Ferulic acid	15.5 ± 0.2 ^b^	12.6± 0.1 ^d^	20.5 ± 0.1 ^a^	13.5 ± 0.2 ^c,d^	14.3 ± 0.3 ^b,c^

Values are mean ± standard deviation, and means followed by a different letter within the row are significantly different (*p* ≤ 0.05); values within the brackets show % increase or reduction in respective phenolic compounds. Key: *PPD0* = non-fermented papaya puree at 0 days; *PPD7*: non-fermented papaya puree stored at 4 °C for 7 d; L56*D7: papaya puree fermented with *Leu. pseudomesenteroides* 56 stored at 4 °C for 7 d; W64*D7: papaya puree fermented with *W. cibaria* 64 stored at 4 °C for 7 d; L75*D7: papaya puree fermented with *Lpb. plantarum* 75 and stored at 4 °C for 7 d.

**Table 4:** Influence of fermentation on simulated *in vitro* gastrointestinal digestion of different phenolic compounds in papaya puree (mg/kg)**.**

Phenolic compounds in papaya puree (mg/kg)																
	*PPD7*				L56*D7				W64*D7				L75*D7			
Compounds	BD	GP	IP	DP	BD	GP	IP	DP	BD	GP	IP	DP	BD	GP	IP	DP
Total phenol	303.9 ± 1.3 ^g^	407.4 ± 1.7 ^de^	396.3 ± 2.3 ^e^	135.4 ± 0.3 ^i^	294.7 ± 0.1 ^g^	481.3 ± 0.4 ^b^	411.3 ± 0.1 ^d^	400.1 ± 1.1 ^e^	380.0 ± 0.3 ^f^	468.2 ± 0.1 ^c^	367.4 ± 1.3 ^f^	273.4 ± 0.5 ^h^	395.4 ± 0.4 ^e^	502.4 ± 0.4 ^a^	372.0 ± 2.4 ^f^	371.3 ± 0.6 ^f^
FRAP (µmol TEAC/100 g FW)	1.2 ± 0.1 ^ij^	3.7 ± 0.1 ^cd^	3.5 ± 1.1 ^d^	1.1 ± 1.0 ^j^	2.0 ± 0.3 ^h^	2.9 ± 0.2 ^e^	2.5 ± 0.7 ^f^	1.4 ± 0.0 ^i^	2.7 ± 0.5 ^ef^	3.9 ± 1.6 ^bc^	4.0 ± 0.1 ^b^	2.0 ± 0.3 ^h^	2.8 ± 0.2 ^e^	4.7 ± 1.0 ^a^	3.7 ± 1.2 ^c^	2.3 ± 0.1 ^g^
Gallic acid	6.7± 1.5 ^f^	6.1 ± 0.2 ^f^	185.0 ± 1.0 ^a^	73.7 ± 2.6 ^d^	5.6 ± 0.1 ^f^	140.2 ± 2.1 ^b^	150.9 ± 13.5 ^b^	47.6 ± 0.1 ^e^	2.9 ± 0.2 ^f^	89.2 ± 1.2 ^d^	126.8 ± 0.6 ^c^	42.9 ± 6.0 ^e^	6.4 ± 0.5 ^f^	57.5 ± 0.4 ^e^	139.6 ± 0.2 ^bc^	43.2 ± 1.3 ^e^
Gallocatechin gallate	564.2 ± 1.8 ^a^	534.6 ± 7.2 ^b^	264.3 ± 4.4 ^f^	32.9 ± 7.6 ^ij^	218.9 ± 2.4 ^f^	216.3 ± 1.1 ^f^	100.4 ± 0.7 ^h^	16.6 ± 1.3 ^j^	462.5 ± 9.8 ^c^	447.3 ± 7.4 ^c^	158.6 ± 2.3 ^g^	33.2 ± 1.1 ^ij^	330.4 ± 1.9 ^d^	311.7 ± 22.2 ^e^	111.4 ± 9.5 ^h^	47.8 ± 4.4 ^i^
Protocatechuic acid	19.3± 0.6 ^f^	18.5 ± 0.4 ^fg^	15.4 ± 0.5 ^fg^	8.9 ± 0.2 ^g^	44.4 ± 2.0 ^c^	32.1 ± 0.4 ^de^	39.8 ± 1.8 ^cd^	15.1 ± 0.1 ^fg^	19.3 ± 0.8 ^g^	39.4 ± 0.7 ^cd^	66.1 ± 6.0 ^a^	24.7 ± 0.6 ^e^	17.4 ± 0.1 ^fg^	29.3 ± 2.7 ^e^	56.6 ± 0.3 ^b^	18.4 ± 0.2 ^fg^
Catechin	14.7 ± 1.5 ^h^	13.4 ± 1.1 ^h^	26.6 ± 3.5 ^gh^	5.5 ± 0.2 ^h^	66.1 ± 2.7 ^de^	139.2 ± 5.5 ^c^	211.4 ± 9.8 ^b^	39.5 ± 1.2 ^fg^	58.2 ± 0.7 ^ef^	84.2 ± 6.4 ^d^	137.4 ± 7.9 ^c^	41.1 ± 2.3 ^fg^	51.7 ± 1.3 ^ef^	121.9 ± 5.4 ^c^	277.5 ± 6.6 ^a^	68.3 ± 2.7 ^de^
Chlorogenic acid	17.2± 0.4 ^b^	22.1 ±0.2 ^a^	8.0 ± 0.1 ^c^	2.2 ± 0.1 ^e^	17.7 ± 0.2 ^b^	3.5 ± 1.4 ^e^	7.6 ± 0.1 ^c^	0.7 ± 0.2 ^e^	1.3 ± 0.8 ^ef^	5.9 ± 2.2 ^d^	10.0 ± 3.3 ^c^	4.8 ± 0.4 ^de^	1.7 ± 0.2 ^e^	5.6 ± 1.0 ^d^	10.0 ± 0.1 ^c^	4.2 ± 0.2 ^de^
Vanillic acid	4.5± 0.8 ^c^	4.9 ± 0.5 ^c^	2.5 ± 0.1 ^cd^	1.1 ± 0.3 ^d^	4.5 ± 0.4 ^c^	4.1 ± 0.8 ^c^	4.8 ± 0.2 ^c^	2.1 ± 0.1 ^cd^	2.5 ± 0.5 ^cd^	5.4 ± 2.0 ^bc^	7.5 ± 0.3 ^bc^	2.5 ± 0.1 ^cd^	2.5 ± 1.1 ^cd^	9.7 ± 0.9 ^ab^	12.7 ± 0.2 ^a^	2.5 ± 0.3 ^cd^
Syringic acid	4.9± 0.1 ^e^	5.1 ± 1.2 ^e^	17.3 ± 0.4 ^cd^	8.2 ± 1.0 ^e^	7.2 ± 0.4 ^e^	7.9 ± 1.6 ^e^	20.3 ± 1.5 ^c^	13.5 ± 0.8 ^d^	2.5 ± 0.1 ^ef^	3.7 ± 0.6 ^ef^	19.3 ± 0.9 ^cd^	3.4 ± 0.1 ^ef^	4.8 ± 1.2 ^ef^	35.2 ± 1.4 ^a^	29.3 ± 0.9 ^b^	1.2 ± 1.2 ^f^
Ellagic acid	5.4± 1.1 ^d^	5.6 ± 0.3 ^cd^	11.2 ± 0.7 ^c^	8.1 ± 0.7 ^cd^	6.2 ± 1.5 ^cd^	4.3 ± 2.0 ^d^	13.3 ± 0.9 ^bc^	7.1 ± 0.1 ^cd^	3.1 ± 0.2 ^d^	4.8 ± 1.3 ^d^	12.2 ± 0.4 ^bc^	6.2 ± 0.4 ^cd^	12.4 ± 0.2 ^bc^	19.3 ± 2.4 ^b^	29.1 ± 0.7 ^a^	2.8 ± 0.6 ^d^
Quercetin	103.8± 16.3 ^a^	87.5 ± 2.3 ^b^	62.8 ± 0.9 ^c^	38.1 ± 0.8 ^f^	38.1 ± 1.5 ^f^	31.7 ± 1.7 f^g^	27.4 ± 2.9 ^gh^	15.9 ± 0.4 ^i^	52.8 ± 5.0 ^e^	58.1 ± 2.4 ^de^	63.2 ± 3.1 ^cd^	25.9 ± 0.8 ^g^	57.7 ± 2.3 ^de^	68.9 ± 2.1 ^bc^	73.5 ± 3.1 ^b^	18.8 ± 2.3 ^hi^
p-Coumaric acid	23.7± 2.5 ^ef^	21.6 ± 0.7 ^ef^	113.8 ± 10.0 ^a^	54.4 ± 0.9 ^c^	37.4 ± 3.2 ^d^	32.5 ± 0.6 ^de^	29.0 ± 1.6 ^de^	16.7 ± 0.5 ^f^	17.3 ± 0.8 ^f^	74.2 ± 1.6 ^c^	87.2 ± 0.7 ^b^	24.0 ± 5.8 ^ef^	25.4 ± 0.8 ^ef^	58.1± 3.7 ^c^	84.4 ± 2.7 ^b^	17.5 ± 0.9 ^f^
Ferulic acid	12.6± 0.1 ^f^	13.4 ± 0.9 ^ef^	15.8 ± 0.2 ^e^	6.0 ± 0.1 ^g^	20.5 ± 0.1 ^bc^	18.9 ± 1.1 ^cd^	17.2 ± 0.5 ^de^	11.5 ± 0.3 ^f^	13.5 ± 0.^ef^	15.0 ± 0.8 ^e^	18.9 ± 0.1 ^cd^	7.0 ± 0.7 ^g^	14.3 ± 0.3 ^e^	19.2 ± 1.1 ^c^	27.4 ± 0.4 ^a^	12.1 ± 0.2 ^f^

Values are mean ± standard deviation, and means followed by a different letter within the row are significantly different (*p* ≤ 0.05). BD: before digestion; GP: gastric phase; IP: intestinal phase; DP: dialysis phase; DW: dry weight; *PPD7*: non-fermented papaya puree stored at 4 °C for 7 d; L56*D7: papaya puree fermented with *Leuconostoc pseudomesenteroides* 56 stored at 4 °C for 7 d; W64*D7: papaya puree fermented with *Weissella cibaria* 64 stored at 4 °C for 7 d; L75*D7: papaya puree fermented with *Lpb. plantarum* 75 and stored at 4 °C for 7 d.

**Table 5:** Recovery and bioaccessibility (%) of different phenolic compounds in fermented and non-fermented papaya puree.

	*PPD7*			L56*D7			W64*D7			L75*D7		
	Recovery%		Bioaccessibility%	Recovery%		Bioaccessibility%	Recovery%		Bioaccessibility%	Recovery%		Bioaccessibility%
Phenolic Compounds	GP	IP	DP	GP	IP	DP	GP	IP	DP	GP	IP	DP
Gallic acid	91.0 ± 3.0 ^i^	2761.2 ± 5.7 ^c^	1100.0 ± 6.2 ^f^	2503.6 ± 6.0 ^c^	2694.6 ± 3.9 ^c^	850.0 ± 3.0 ^g^	3075.9 ± 1.8 ^b^	4372.4 ± 5.9 ^a^	1479.3 ± 2.8 ^e^	898.4 ± 2.9 ^g^	2181.3 ± 3.3 ^d^	675.0 ± 1.5 ^h^
Gallocatechin gallate	94.8 ± 1.0 ^ab^	46.8 ± 0.8 ^b^	5.8 ± 0.4 ^f^	98.8 ± 1.9 ^a^	45.9 ± 2.0 ^b^	7.6 ± 0.4 ^e^	96.7 ± 2.5 ^a^	34.3 ± 1.9 ^c^	7.2 ± 0.5 ^e^	94.3 ± 1.7 ^ab^	33.7 ± 2.4 ^c^	14.5 ± 1.7 ^d^
Protocatechuic acid	95.9 ± 2.1 ^e^	79.8 ± 3.0 ^f^	46.1 ± 2.2 ^h^	72.3 ± 2.4 ^g^	89.6 ± 3.1 ^e^	34.0 ± 0.8 ^i^	204.1 ± 3.1 ^b^	342.5 ± 2.8 ^a^	128.0 ± 1.9 ^cd^	168.4 ± 2.9 ^c^	325.3 ± 2.0 ^ab^	105.7 ± 3.3 ^d^
Catechin	91.2 ± 1.7 ^g^	181.0 ± 2.0 ^d^	37.4 ± 0.5 ^j^	210.6 ± 1.8 ^cd^	319.8 ± 3.4 ^b^	59.8 ± 3.7 ^i^	144.7 ± 2.9 ^e^	236.1 ± 2.2 ^c^	70.6 ± 2.9 ^h^	235.8 ± 3.6 ^c^	536.8 ± 1.8 ^a^	132.1 ± 2.0 ^f^
Epicatechin	87.1 ± 2.1 ^e^	164.5 ± 2.7 ^b^	59.7 ± 0.6 ^f^	86.4 ± 1.0 ^e^	114.8 ± 2.1 ^c^	32.5 ± 0.8 ^h^	109.1 ± 1.5 ^d^	137.7 ± 1.6 ^bc^	40.3 ± 0.8 ^g^	140.7 ± 1.9 ^bc^	207.4 ± 2.2 ^a^	79.6 ± 1.6 ^e^
Caffeic acid	89.1 ± 0.8 ^b^	100.0 ± 2.9 ^a^	28.3 ± 1.2 ^e^	37.1 ± 1.1 ^d^	43.8 ± 0.9 ^c^	12.4 ± 0.5 ^f^	97.8 ± 2.0 ^ab^	100.0 ± 2.3 ^a^	37.0 ± 4.0 ^d^	93.5 ± 1.5 ^b^	100.0 ± 2.0 ^a^	106.5 ± 3.0 ^a^
Chlorogenic acid	128.5 ± 1.1 ^e^	46.5 ± 0.8 ^f^	12.8 ± 0.1 ^h^	19.8 ± 0.9 ^g^	42.9 ± 0.8 ^f^	4.0 ± 0.1 ^i^	453.8 ± 2.1 ^bc^	769.2 ± 1.8 ^a^	369.2 ± 3.1 ^c^	329.4 ± 2.4 ^c^	588.2 ± 1.5 ^b^	247.1 ± 0.7 ^d^
Vanillic acid	108.9 ± 3.3 ^d^	55.6 ± 2.0 ^f^	24.4 ± 0.7 ^h^	91.1 ± 2.9 ^e^	106.7 ± 1.0 ^d^	46.7 ± 2.2 ^g^	216.0 ± 0.9 ^c^	300.0 ± 3.4 ^bc^	100.0 ± 0.9 ^d^	388.0 ± 2.0 ^b^	508.0 ± 1.0 ^a^	100.0 ± 0.4 ^d^
Syringic acid	104.1 ± 2.9 ^g^	353.1 ± 2.7 ^c^	167.3 ± 2.0 ^e^	109.7 ± 3.1 ^g^	281.9 ± 1.2 ^d^	187.5 ± 4.5 ^de^	148.0 ± 3.4 ^f^	772.0 ± 6.0 ^a^	136.0 ± 1.8 ^f^	733.3 ± 3.0 ^ab^	610.4 ± 1.9 ^b^	25.0 ± 0.5 ^h^
Ellagic acid	103.7 ± 2.9 ^f^	207.4 ± 0.8 ^c^	150.0 ± 1.9 ^e^	69.4 ± 1.7 ^g^	214.5 ± 1.1 ^c^	114.5 ± 0.7 ^f^	154.8 ± 1.2 ^e^	393.5 ± 2.0 ^a^	200.0 ± 1.9 ^d^	155.6 ± 2.1 ^e^	234.7 ± 2.8 ^b^	22.6 ± 0.9 ^h^
Quercetin	84.3 ± 2.0 ^c^	60.5 ± 1.1 ^d^	36.7 ± 0.6 ^f^	83.2 ± 1.4 ^c^	71.9 ± 2.2 ^cd^	41.7 ± 0.7 ^e^	110.0 ± 2.8 ^b^	119.7 ± 1.6 ^ab^	49.1 ± 0.1 ^e^	119.4 ± 2.0 ^ab^	127.4 ± 1.9 ^a^	32.6 ± 2.2 ^f^
p-Coumaric acid	91.1 ± 2.0 ^f^	480.2 ± 2.7 ^ab^	229.5 ± 3.5 ^d^	86.9 ± 2.5 ^f^	77.5 ± 1.8 ^g^	44.7 ± 2.6 ^h^	428.9 ± 4.1 ^b^	504.0 ± 2.1 ^a^	138.7 ± 1.9 ^e^	228.7 ± 2.8 ^d^	332.3 ± 3.3 ^c^	68.9 ± 1.7 ^g^
Ferulic acid	106.3 ± 1.3 ^d^	125.4 ± 0.9 ^c^	47.6 ± 1.2 ^h^	92.2 ± 2.5 ^e^	83.9 ± 0.9 ^e^	56.1 ± 1.3 ^f^	111.1 ± 0.9 ^d^	140.0 ± 2.8 ^b^	51.9 ± 3.6 ^g^	134.3 ± 1.9 ^b^	191.6 ± 2.1 ^a^	84.6 ± 3.5 ^e^

Values are mean ± standard deviation, and means followed by a different letter within the row are significantly different (*p* ≤ 0.05). BD: before digestion; GP: gastric phase; IP: intestinal phase; DP: dialysis phase; DW: dry weight; *PPD7*: non-fermented papaya puree stored at 4 °C for 7 d; L56*D7: papaya puree fermented with *Leu. pseudomesenteroides* 56 stored at 4 °C for 7 d; W64*D7: papaya puree fermented with *W. cibaria* 64 stored at 4 °C for 7 d; L75*D7: papaya puree fermented with *Lpb. plantarum* 75 and stored at 4 °C for 7 d.

## Data Availability

Data is contained within the article or Supplementary Materials. The data presented in this study are available in the article and also in supplementary file Table S1 attached in the manuscript.

## Supplementary Materials

The following are available online at https://www.mdpi.com/article/10.3390/foods10050962/s1, Table S1: The identification of different phenolic compounds.
