# Supplementary material for: Influence of Fermentation of Pasteurised Papaya Puree with Different Lactic Acid Bacterial Strains on Quality and Bioaccessibility of Phenolic Compounds during In Vitro Digestion
Source: Foods. 2021 Apr 28;10(5):962. doi: 10.3390/foods10050962 (PMC8145966; doi:10.3390/foods10050962)
Supplement: Supplementary file 1 [file foods-10-00962-s001.zip › foods-1181630-supplementary.pdf]

Changes in phenolics and antioxidant capacity during simulated *in vitro* digestion of papaya puree fermented with different lactic acid bacteria

Florence M. Mashitoa[a,c], Cyrielle Garcia[b], Fabienne Remize[b], Vimbainashe E. Manhivi[a], Stephen A Akinola[a], Reatha M. Slabbert[c], Dharini Sivakumar[a]

Supplementary Table 1 The identification of different phenolic compounds

Phenolic compounds	Retention time (mins)	Regression equation	R^2	LOD (μg /l)	LOQ (μg /l)
gallic	7.897	y =99324x+55626	0.998	0.05	0.186
Gallocatechin (catechin)	10.67	Y=2828x-69172	0.999	3.2	5.7
Protochatechuic	11.255	y=19722x-341718	0.998	3.2	10.9
Catechin	13.24	Y=2828x-69172	0.999	3.2	15.7
Epicatechin	14.2	Y=35316x+193517	0.997	1.4	3.3
vanillic	14.907	y=39979x-125065	0.997	2.2	7.3
chlorogenic	14.724	y=71930x-2110,5	0.995	0.11	0.37
Quercetin	16.77	y=12091x-170181	0.996	7.7	25.8
Ferulic	17. 93	y=20067x-279209	0.999	3.3	11
p-coumaric	17.54	y=6925.3x-62646	0.999	10.25	34.17
Syringic	15.44	y=75813x-107617	0.999	0.05	0.18
Ellagic	15. 996	y=20110x+9484.9	0.997	0.31	1
